# Reconstruction and analysis of the genome-scale metabolic model of *schizochytrium limacinum* SR21 for docosahexaenoic acid production

**DOI:** 10.1186/s12864-015-2042-y

**Published:** 2015-10-16

**Authors:** Chao Ye, Weihua Qiao, Xiaobin Yu, Xiaojun Ji, He Huang, Jackie L. Collier, Liming Liu

**Affiliations:** State Key Laboratory of Food Science and Technology, Jiangnan University, 1800 Lihu Road, Wuxi, Jiangsu 214122 China; The Key Laboratory of Industrial Biotechnology, Ministry of Education, Jiangnan University, 1800 Lihu Road, Wuxi, Jiangsu 214122 China; State Key Laboratory of Materials-Oriented Chemical Engineering, College of Biotechnology and Pharmaceutical Engineering, Nanjing Tech University, No. 30 South Puzhu Road, Nanjing, 211816 China; School of Marine and Atmospheric Sciences, Stony Brook University, Stony Brook, NY USA

**Keywords:** *Schizochytrium limacinum* SR21, Docosahexaenoic acid, Genome-scale metabolic model, Polyketide synthase system, Minimization of metabolic adjustment algorithm

## Abstract

**Background:**

*Schizochytrium limacinum* SR21 is a potential industrial strain for docosahexaenoic acid (DHA) production that contains more than 30–40 % DHA among its total fatty acids.

**Methods:**

To resolve the DHA biosynthesis mechanism and improve DHA production at a systematic level, a genomescale metabolic model (GSMM), named iCY1170_DHA, which contains 1769 reactions, 1659 metabolites, and 1170 genes, was reconstructed.

**Results:**

Based on genome annotation results and literature reports, a new DHA synthesis pathway based on a polyketide synthase (PKS) system was detected in *S. limacinum*. Similarly to conventional fatty acid synthesis, the biosynthesis of DHA via PKS requires abundant acetyl-CoA and NADPH. The *in silico* addition of malate and citrate led to increases of 24.5 % and 37.1 % in DHA production, respectively. Moreover, based on the results predicted by the model, six amino acids were shown to improve DHA production by experiment. Finally, 30 genes were identified as potential targets for DHA over-production using a Minimization of Metabolic Adjustment algorithm.

**Conclusions:**

The reconstructed GSMM, *i*CY1170_DHA, could be used to elucidate the mechanism by which DHA is synthesized in *S. limacinum* and predict the requirements of abundant acetyl-CoA and NADPH for DHA production as well as the enhanced yields achieved via supplementation with six amino acids, malate, and citrate.

**Electronic supplementary material:**

The online version of this article (doi:10.1186/s12864-015-2042-y) contains supplementary material, which is available to authorized users.

## Background

Docosahexaenoic acid (DHA), which is an n-3 polyunsaturated fatty acid (PUFA), has been shown to have a positive effect on diseases such as hypertension, arthritis, atherosclerosis, depression, adult-onset diabetes mellitus, myocardial infarction, thrombosis, and some cancers [1]. DHA is necessary for the development of the brain and retina of infants, and it is important in maintaining brain function in adults [2]. Oceanic fish and fish oil products are typical dietary sources of DHA [3]. However, because of emerging concerns over the sustainability of marine resources and the levels of environmental contaminants present in fish, major efforts have been made to identify or create alternative sources of DHA [4].

*Schizochytrium limacinum* SR21 (*Aurantiochytrium limacinum* ATCC MYA-1381) is a marine thraustochytrid that can synthesize lipids with a high content of DHA. In SR21, total fatty acids reportedly constitute more than 50 % of the dry cell weight (DCW) [5], and about 30–40 % of the fatty acids of this strain are DHA [6]. In addition, a number of studies have confirmed the safety of DHA-rich oil extracted from *Schizochytrium* sp. in recent years [7, 8]. For these reasons, DHA from microorganisms has been widely incorporated into infant formulas and health products for elderly persons [9]. In addition, industrial utilization of this organism as a commercial source of DHA is currently receiving much attention [10, 11].

The dry cell weight, lipid content, and DHA percentage of the total fatty acids are three important parameters for evaluating fermentations. Many efforts have been directed towards improving the DHA yield. (1) To optimize the culture medium, it was demonstrated that using glucose and glycerol as mixed carbon sources, the DHA productivity was 15.24 % higher than that obtained using glucose as single carbon source [12]. (2) To improve the fermentation process, a NH_4_-pH-auxostat system was developed that appears to be a promising technique for the first stage of production of *Schizochytrium* sp. biomass as a means of achieving the fastest possible growth rate [13]. A two-stage oxygen supply control strategy was applied to the DHA fermentation. With this protocol, the production of biomass and DHA improved to 37.9 g/L and 6.56 g/L, which increased 18.1 % and 9.88 %, respectively [10]. (3) To enhance metabolic regulation, a strategy was proposed that reinforces acetyl-CoA and NADPH supply. By adding 4 g/L malic acid, the DHA content among the total fatty acids increased from 35 % to 60 %. The total lipid content also showed an apparent increase of 35 % and reached 19 g/L when 40 mL ethanol/L were added [14]. Although many strategies have been applied to improve DHA production, some problems still exist during the fermentation process. For example, the growth of SR21 is unstable and cell viability is low. The mechanism of DHA synthesis is still unclear, and cell metabolism is difficult to regulate by genetic manipulation.

A genome-scale metabolic model (GSMM) represents the microbial metabolic genotype–phenotype relationships of an organism [15]. Such models have been widely used in many contexts, such as contextualizing high-throughput data, understanding complex biological phenomena, guiding metabolic engineering, directing hypothesis-driven discovery, interrogating multi-species relationships, and discovering network properties [16–19]. The release of the whole genome sequence of *S. limacinum* SR21 and corresponding literature reports have made the reconstruction of a GSMM possible.

In this study, a GSMM of *S. limacinum* SR21 was reconstructed. Using this model, we first made a comparison with two oleaginous fungi, *Mortierella alpina* and *Yarrowia lipolytica*, that are used for industrial production of arachidonic acid (ARA) and eicosapentaenoic acid (EPA), respectively. Then the pathway of DHA biosynthesis in SR21 was resolved based on genome annotation results and literature mining. Based on the GSMM, biochemical and genetic strategies were applied to improve DHA production.

## Results and discussion

### Genome-scale reconstruction and general features of model *i*CY1170_DHA

**Fig. 1:**
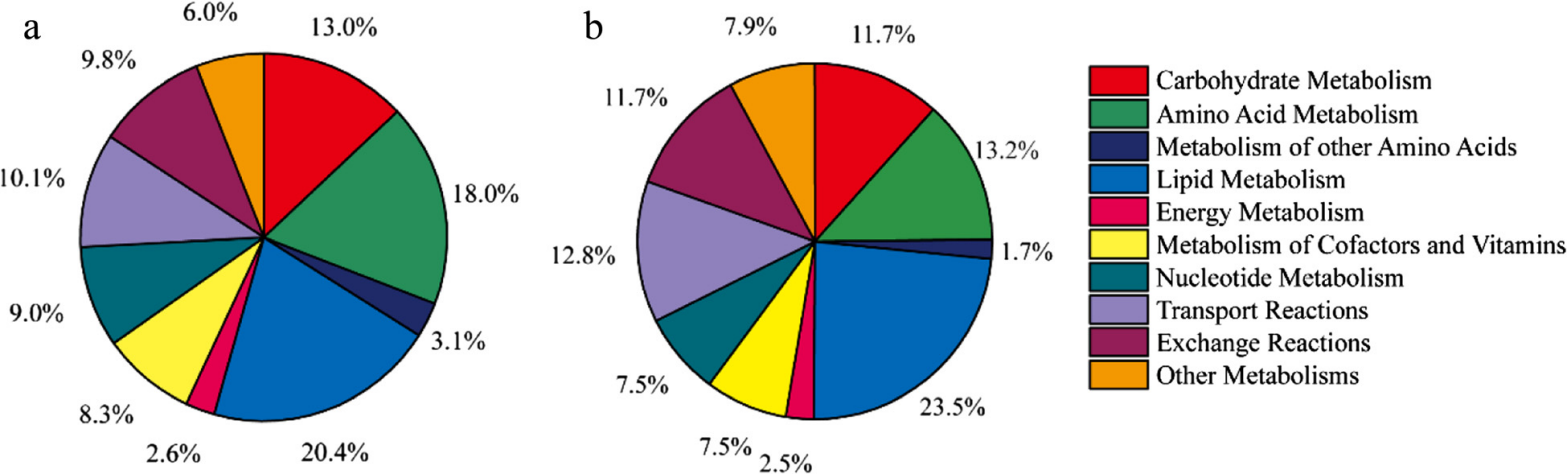
Metabolic subsystems distribution for *in silico* models. **a**, *S limacinum* model *i*CY1170_DHA, **b**, *M. alpina* model *i*CY1106. ‘Other metabolisms’ subsystem contains Glycan Biosynthesis and Metabolism, Metabolism of Terpenoids and Polyketides, Biosynthesis of other secondary metabolites, and Xenobiotics Biodegradation and Metabolism

The reconstructed GSMM of *Schizochytrium limacinum* SR21, which contains 1769 reactions and 1659 metabolites, was named *i*CY1170_DHA (Additional file 1). Model *i*CY1170_DHA consists of 1386 intracellular metabolic reactions and 195 transport reactions. 7.9 % of the total open reading frames (ORFs), corresponding to 1170 genes of 14,859 ORFs, were incorporated into the model. In *i*CY1170_DHA, all 1769 reactions (including transport and exchange reactions) were classified into 10 different subsystems, according to the KEGG Pathway Database (http://www.genome.jp/kegg/pathway.html): carbohydrate metabolism, amino acid metabolism (20 common acid acids), other amino acids (D-type amino acids) metabolism, nucleotide metabolism, energy metabolism, lipid metabolism, metabolism of cofactors and vitamins, transport reactions, exchange reactions, and other metabolisms (Fig. 1a). Among them, lipid metabolism ranks as the largest subsystem in *i*CY1170_DHA (20.4 %), followed by amino acid metabolism (18.0 %), which agrees with model *i*CY1106 for *Mortierella alpina* (Fig. 1b). The sum of the three largest subsystems, lipid metabolism, amino acid metabolism, and carbohydrate metabolism, accounts for over half of the total number of reactions (51.4 %). The difference between the two models was that, in model *i*CY1170_DHA, the third largest subsystem is carbohydrate metabolism, while in model *i*CY1106, transport reactions rank third, which means that *M. alpina* has more transport mechanisms than *S. limacinum*.

**Fig. 2:**
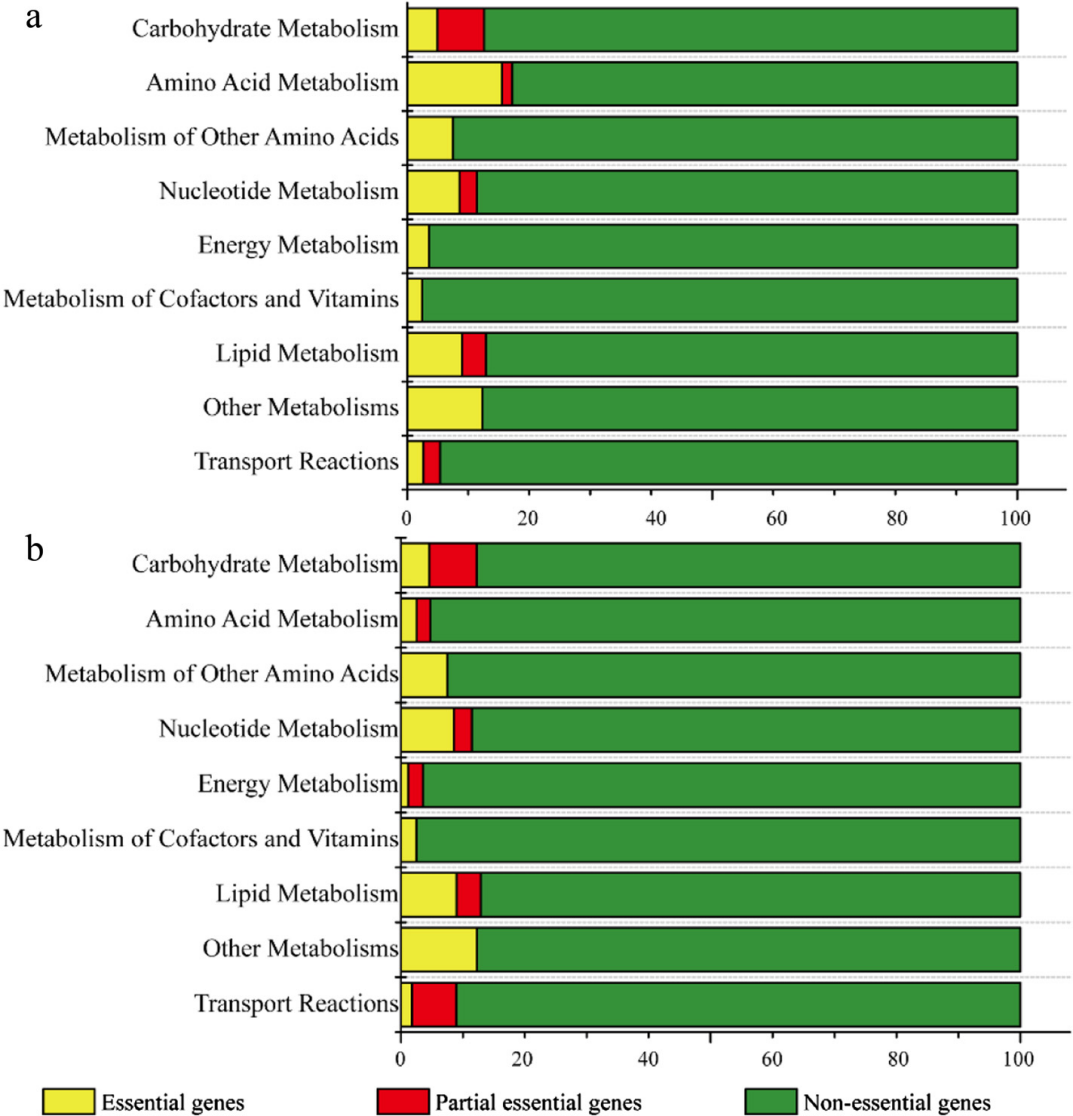
The distribution of essential genes in different media. **a** the minimal glucose medium (MG); **b** the yeast extract medium (YE)

The essentialities of individual genes of *S. limacinum* were analyzed under minimal glucose (MG) and yeast extract (YE) medium conditions using *i*CY1170_DHA by deleting each gene in turn. The genes were categorized into three classes: essential genes, partially essential genes and non-essential genes. The gene essentiality study revealed that a total of 56 genes were essential in both YE and MG medium. An additional 35 genes were essential only in MG medium (Additional file 2). When comparing the distribution of essential genes in different culture media, for amino acid metabolism most genes were only essential in MG medium (increased from 2.6 % to 15.5 %, Fig. 2a). Since YE medium is supplemented with all of the amino acids, some of the amino acids were directly consumed from the medium without utilizing their biosynthetic pathways (Fig. 2b). For example, Aurli1_48454 (argininosuccinate synthase, EC: 6.3.4.5) and Aurli1_69076 (argininosuccinate lyase, EC: 4.3.2.1), which convert aspartate into arginine, were essential only when grown in MG medium. Interestingly, the ‘other metabolism’ subsystem contained the same proportion of essential genes (11.9 % of total reactions in the other metabolism subsystem) in both media. That means that, in this subsystem, the pathway of squalene biosynthesis does not have many alternative routes and is quite rigid in *S. limacinum*.

Similarly to model *i*CY1106, model *i*CY1170_DHA had more reactions, metabolites, and genes than model *i*YL619_PCP [20] for *Yarrowia lipolytica* (Table 1). However, the ORF coverage of *i*CY1170_DHA was a little lower than the other two models. When ignoring compartment information, not including transport and exchange reactions, models *i*CY1170_DHA, *i*CY1106, and *i*YL619_PCP contain 1195, 1124, and 748 reactions, respectively. And 457 reactions were present in all these three models (Additional file 3, Fig. 3). Models *i*CY1170_DHA and *i*CY1106 both shared 803 reactions, which account for 67.2 % and 71.4 % of total reactions, respectively. This means these two models are similar in biochemical reactions to some degree. However, 343 reactions (28.7 % of total reactions) were unique in model *i*CY1170_DHA, and 61.3 % of these unique reactions were distributed across lipid metabolism, amino acid metabolism, and carbohydrate metabolism. For example, it was reported that *S. limacinum* could grow with arabinose as the carbon source [21, 22], whereas *Y. lipolytica* and *M. alpina* could not use arabinose [20]. Reactions that can convert arabinose into glucose (involving 12 reactions) are thus unique in model *i*CY1107_DHA (Additional file 1). In addition, there were two pathways for synthesizing lysine, starting from aspartate or 2-oxoglutarate according to the genome annotation results, whereas in models *i*YL619_PCP and *i*CY1106 only the second pathway for lysine biosynthesis is present. Reactions involving the first pathway must also be unique in model *i*CY1170_DHA.

**Table 1:** Features of the *in silico* genome-scale metabolic model of *S. limacinum*, *M. alpina* and *Y. lipolytica*

Features	*i*CY1170_DHA	*i*CY1106	*i*YL619_PCP
Genome feature			
Genome size (Mb)	60.93	38.38	20.50
Open reading frames (ORFs)	14,859	11,631	6453
*In silico* metabolic model			
Reactions included in the model	1769	1854	1142
Biochemical reactions	1386	1391	781
Transport reactions	195	247	236
Exchange reactions	188	216	125
Metabolites	1659	1732	843
ORFs assigned in metabolic model	1170	1106	619
ORF coverage^a^ (%)	7.9	9.5	9.6

^a^The number of ORFs in the *i*CY1170_DHA model divided by the total number of ORFs in the genome

**Fig. 3:**
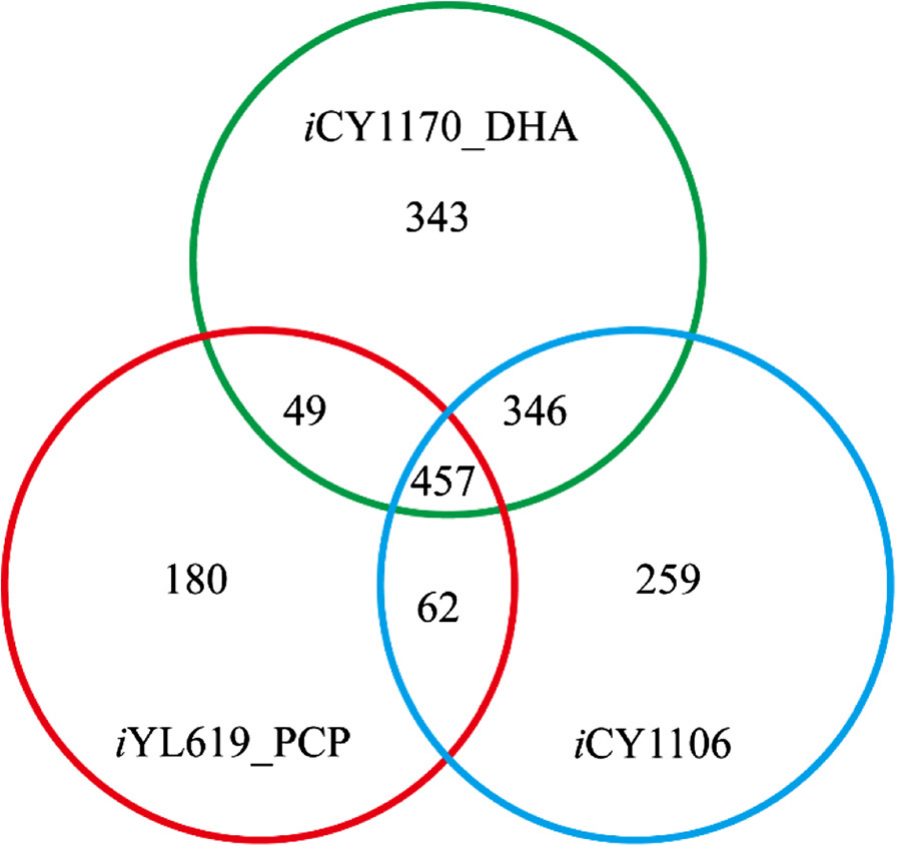
Comparison among three existing models of different oleaginous fungi, *S. limacinum*, *M. alpina*, and *Y. lipolytica*

### Verification and simulation of model *i*CY1170_DHA

Data from batch cultures of *S. limacinum* grown in glucose and glycerol media were used to validate *i*CY1170_DHA. Both of the *in silico* media contain the basic elements, such as C, N, H, O, P, S (Table 2). When using glucose or glycerol as carbon source, their maximum uptake rates were 1.4 mmol/gDW/h and 1.6 mmol/gDW/h, respectively (Additional file 1) [23]. To simulate cellular growth in the different media, the biomass equation was maximized in the flux analysis simulations. Notably, simulation results were consistent with observed growth rates (Table 3). Without the constraints of production, for the glucose medium, the *in silico* simulation predicted cell growth of 0.0812/h, which was very close to the experimentally observed specific growth rate of 0.0887/h (8.5 %) [24]. And for the glycerol medium, the simulated result was only 1.1 % lower than the experimental result [25]. However, when the DHA synthesis rate was constrained at 0.03 mmol/gDW/h [24], the *in silico* result was 16.42 % lower than experimental result [24]. This means the synthesis of DHA may require other nutrients, apart from amino acids. And the replacement of yeast extract by inorganic nitrogen could lead to these nutritional deficiencies.

**Table 2:** The *in silico* glucose minimal media composition of the model *i*CY1170_DHA

Reaction description	Equation	LB^a1^	UB^b2^
Exchange of D-glucose	D-glucose[e] < =>	−1.4	1000
Exchange of Water	H_2_O[e] < =>	−1000	1000
Exchange of Oxygen	O_2_[e] < =>	−1000	1000
Exchange of Ammonia	NH_3_[e] < =>	−10	1000
Exchange of Orthophosphate	Pi[e] < =>	−1000	1000
Exchange of Sulfate	SO_4_[e] < =>	−1000	1000
Exchange of H^+^	H[e] < =>	−1000	1000

^a^1, LB, lower bound, whose unit is mmol/gDW/h. ^b^2, UB, upper bound, whose unit is also mmol/gDW/h

**Table 3:** Comparison of *in silico* and in vivo growth rates of *S. limacinum*

Media condition	Growth rate (/h)	
(mmol/gDW/h)	*In vivo*	*In silico*
glucose (v = 1.4)	0.0887	0.0812
glucose (v = 1.4, DHA production = 0.03)	0.0883	0.0738
glycerol (v = 1.6)	0.0620	0.0613

*In vivo*: experimental results, *In silico*: simulation results. *In vivo* growth rate was calculated from the growth curve of *S. limacinum. In silico* growth rate was the solution of S (m × n) matrix, when the biomass function was used as objective function

Apart from the growth rate simulation, the ability of *i*CY1170_DHA to utilize different carbon and nitrogen sources has been verified. According to literature reports, *S. limacinum* can grow using 15 kinds of carbon sources and 3 kinds of inorganic nitrogen sources, such as NH_4_^+^, NO_3_^−^, and urea. However, the simulation results show that the model could not grow using 5 carbon sources, including xylose, arabinose, lactose, starch, and trehalose (Table 4). It also could not grow on a medium using NO_3_^−^ as the nitrogen source. After a series of gap filling and model debugging steps, ten reactions were added to the model. For example, lactose galactohydrolase (EC: 3.2.1.23), which can convert lactose into glucose, was not found during genome annotation. After filling this gap, the model could grow with lactose as the carbon source. All of these simulation results, including the growth rate simulations and usage of different carbon and nitrogen sources, indicated that the model *i*CY1170_DHA is reliable and can be used for further prediction and analysis.

**Table 4:** Comparison between experimental results and simulated results about the usage of different carbon and nitrogen sources

Carbon source	*In vivo*	*In silico* ^a^	*In silico* ^b^	Reference
Glucose	+	+	+	[6, 21, 22]
Fructose	+	+	+	[6, 21, 22]
Mannose	+	+	+	[21, 22]
Galactose	+	+	+	[21, 22]
Xylose	+	-	+	[21, 22]
Arabinose	+	-	+	[21, 22]
Ribose	+	+	+	[21, 22]
Lactose	+	-	+	[6, 21, 22]
Sucrose	+	+	+	[21, 22]
Maltose	+	+	+	[6, 21, 22]
Starch	+	-	+	[6, 21, 22]
Glycerol	+	+	+	[6, 21, 22]
Melibiose	+	+	+	[21]
Raffinose	+	+	+	[21]
Trehalose	+	-	+	[21]
**Nitrogen Source**				
NH_4_^+^	+	+	+	[6]
NO_3_^−^	+	-	+	[6]
Urea	+	+	+	[6, 21]

^a^Before filling gaps; ^b^After filling gaps. To make the GSMM consistent with experimental data, some missing reactions were added to model

### Resolving the DHA synthesis pathway based on model *i*CY1170_DHA

**Fig. 4:**
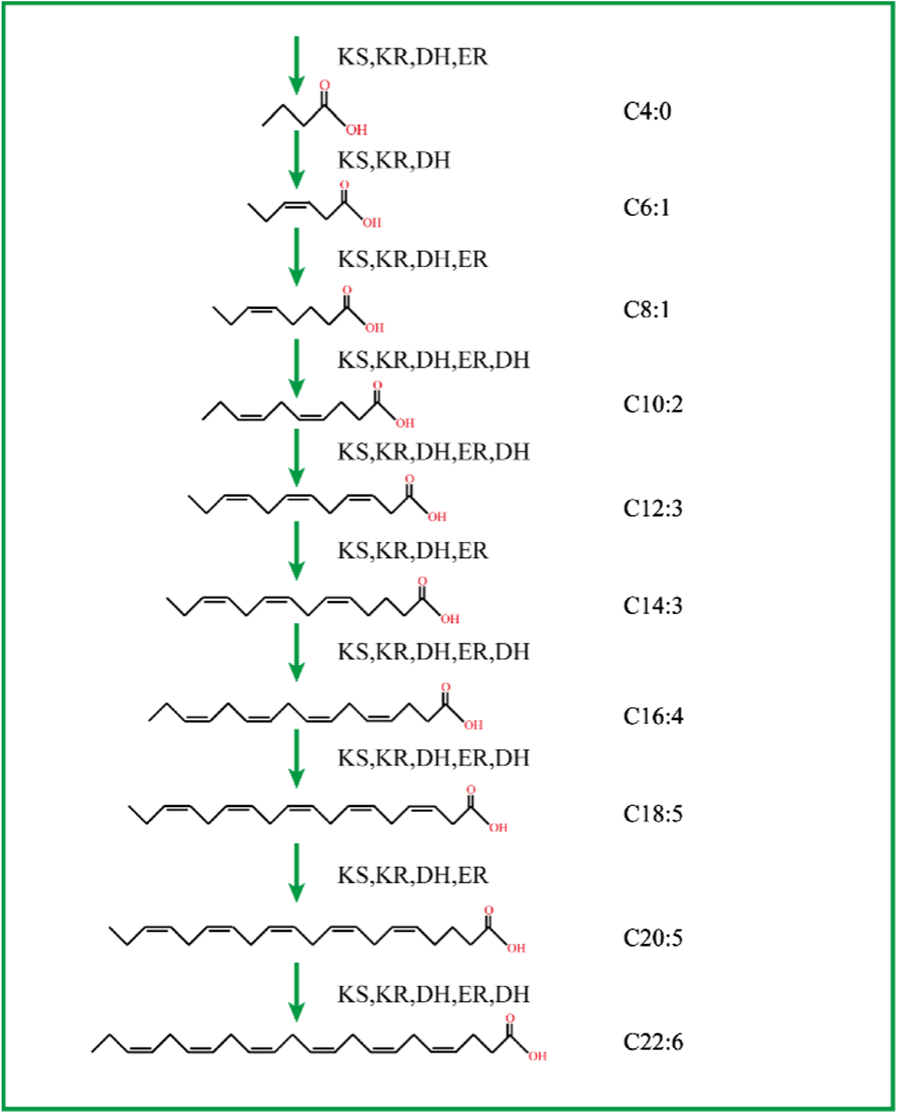
A scheme to account for the formation of DHA by the PKS route of synthesis in *S. limacinum*. KS, 3-ketoacyl synthase; KR, 3-ketoacyl-ACP reductase; ER, enoyl reducatase; DH, dehydrase/isomerase

There are two pathways that can synthesize DHA, the conventional fatty acid synthesis (FAS) route and the polyketide synthase (PKS) system [26]. In the FAS route, fatty acids are biosynthesized in the form of either C16:0 or C18:0 saturated fatty acids. These fatty acids are then modified through a sequence of desaturations and elongations so that extended ranges of unsaturated fatty acids and PUFAs are produced [27]. In this route, DHA is synthesized by delta-4 desaturase, which can transform C22:5 to C22:6 (DHA). In the PKS pathway, acyl carrier protein (ACP), generated by CoA, is used as a covalent attachment point for chain synthesis, which proceeds with reiterative cycles. During the full fatty acid synthesis process, a series of enzymes including 3-ketoacyl synthase (KS), 3-ketoacyl-ACP reductase (KR), enoyl reducatase (ER), and dehydrase/isomerase (DH) are necessary (Fig. 4). However, the whole genome annotation results showed that *S. limacinum* does not contain delta-4 desaturase, in agreement with literature reports. On the other hand, some ORFs in *S. limacinum* were predicted to be potential PKS proteins (Additional file 4). This means *S. limacinum* could not synthesize DHA by the FAS route, but rather employs the PKS system for PUFA biosynthesis [26, 28, 29].

**Fig. 5:**
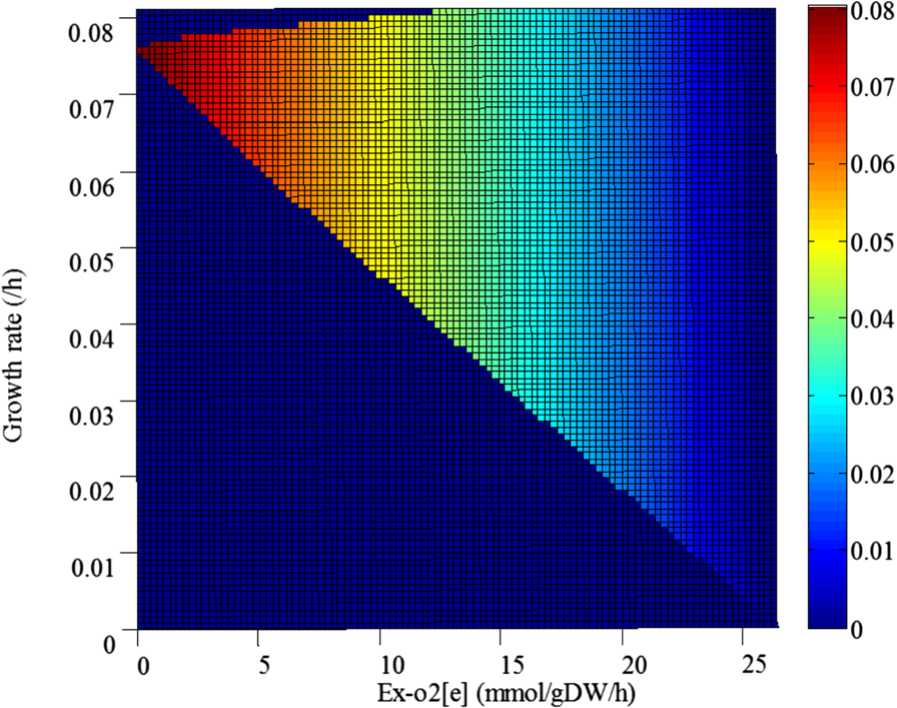
DoubleRobustnessAnalysis of the relationship among oxygen uptake rate, growth rate and DHA production

When compared with *M. alpina*, which synthesizes arachidonic acid via the FAS route, the DHA synthesis in *S. limacinum* does not need much more oxygen in the PKS route. DoubleRobustnessAnalysis results showed that, when the growth rate was fixed, a low oxygen uptake rate (below 5 mmol/gDW/h) was beneficial for DHA accumulation (Fig. 5) while, at the cell-number-increasing stage, to acquire large quantities of cells for lipid accumulation, an abundant oxygen supply was necessary. This means that, during the DHA fermentation process, a two-stage oxygen supply strategy could improve DHA production efficiency [10, 30, 31].

Similarly to the FAS pathway, two steps in the PKS cycle, catalyzed by KR and ER, also require NADPH [29]. Flux balance analysis shows that, in YE medium, a total of 57 reactions involving NADPH have fluxes (Additional file 5). And of these reactions, three are major sources of NADPH. One is catalyzed by malic enzyme (ME, EC: 1.1.1.40). The other two are in the pentose phosphate pathway and are catalyzed by glucose-6-phosphate dehydrogenase (G6PD, EC: 1.1.1.49) and phosphogluconate dehydrogenase (PGD, EC: 1.1.1.44), respectively. Their corresponding fluxes of NADPH were 1.41 mmol/gDW/h, 1.49 mmol/gDW/h, and 1.49 mmol/gDW/h. In addition to NADPH, acetyl-CoA, which is the precursor for fatty acid *de novo* biosynthesis, also plays an important role. The flux of acetyl-CoA used for fatty acid synthesis was 1.88 mmol/gDW/h.

### Biochemical engineering strategies for improving DHA production by *in silico* simulation

Malate plays an important role in the TCA cycle. Catalyzed by ME, malate can be converted into pyruvate, accompanied by NADPH. Based on the YE medium, when the maximum uptake rate of malate was set at 1 mmol/gDW/h, DHA production increased 24.5 %. This was in agreement with Ren’s report that, after adding 4 g/L malate, the DHA content of the total fatty acids increased from 35 % to 60 % [14]. It was also proved that the addition of malate lead to an increase in DHA production of 40.02 % [32]. Flux balance analysis (FBA) results showed that, by adding malate, the flux of NADPH supplied by the reaction catalyzed by ME increased from 1.4 mmol/gDW/h to 1.8 mmol/gDW/h, an increase of 28.6 %. Additionally, flux distribution showed that the pentose phosphate pathway was enhanced by 23.5 %, which means that, by adding malate, more NADPH was supplied for DHA biosynthesis. In addition to NADPH, citrate lyase (ACL, EC: 2.3.3.8), which can catalyze the cleavage of citrate, is the source of acetyl-CoA for fatty acid biosynthesis [33]. Adding citrate *in silico* led to a 37.1 % increase in DHA production. The corresponding acetyl-CoA flux provided by this pathway was also increased by 23.2 %. This also agreed with Wang’s report that a 47.17 % improvement in DHA production was gained by adding citrate [32].

**Fig. 6:**
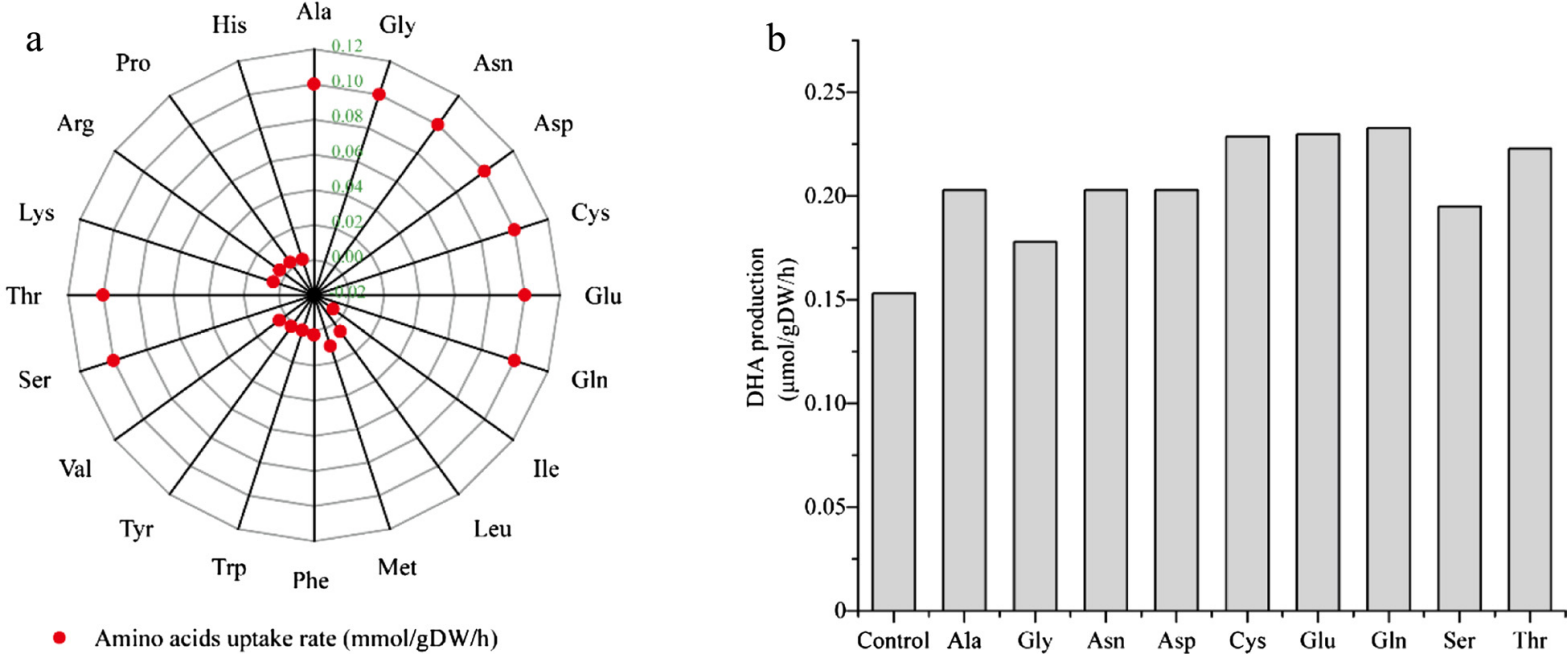
Simulation results of amino acids uptake rates and their effects on DHA production. **a** *In silico* result of 20 different amino acids uptake rates in YE medium. **b** Effect on DHA production by adding different amino acids to MG medium

DCW, lipid content, and DHA percentage of total fatty acids are three important parameters for evaluating the fermentation process. And amino acids can be utilized by microorganisms both as a carbon source and a nitrogen source for cell growth. In YE medium, we first calculated the uptake rate of 20 amino acids by FBA. For nine amino acids (Ala, Gly, Asn, Asp, Cys, Glu, Gln, Ser, and Thr), the uptake rate could reach the set maximum values (Fig. 6a). For the others, the uptake rates were lower than 0.01 mmol/gDW/h. This means these nine amino acids could promote the growth of *S. limacinum* SR21, so the effect of each amino acid on DHA production was simulated by adding them individually to the MG medium. The maximum uptake rate of each amino acid was fixed at 1 mmol/gDW/h, and only the nine amino acids could promote both growth and DHA production. DHA production was increased by 32.7 %, 16.3 %, 32.7 %, 32.7 %, 50.3 %, 50.3 %, 52.3 %, 27.5 %, and 45.8 %, respectively (Fig. 6b).

**Fig. 7:**
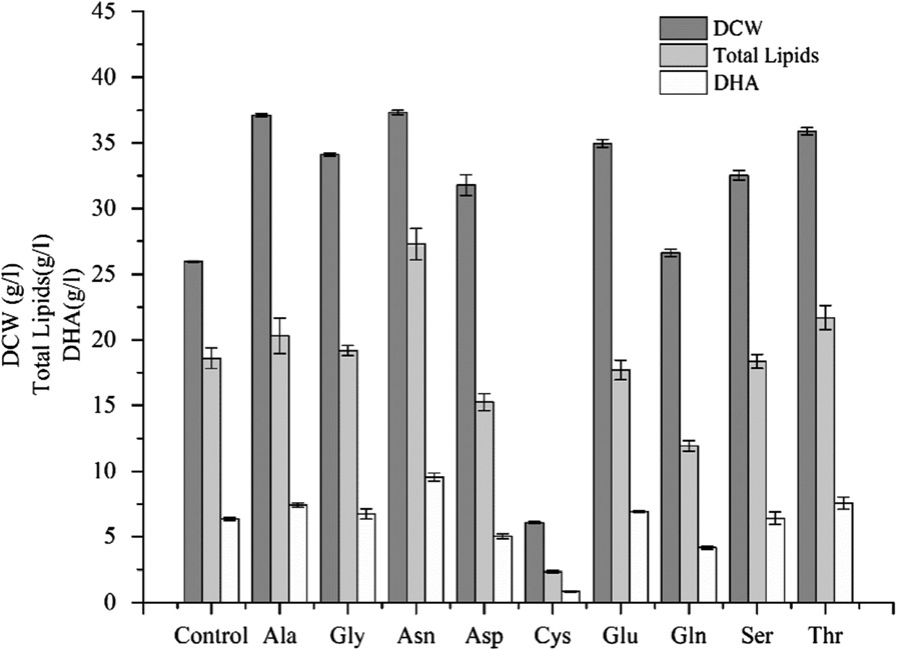
Effects on dry cell weight, total lipids, and DHA production of adding nine different amino acids to *S. limacinum* cultures

Furthermore, these simulation results were verified by experiment. The experimental results showed that addition of eight of the nine amino acids (except cysteine) could promote cell growth (Fig. 7). The addition of asparagine could improve the dry cell weight from 26.0 g/L to 37.4 g/L, an increase of 43.8 %, whereas adding cysteine to the culture medium inhibited the growth of SR21. The reason may be that only cysteine contains sulfur, and excess sulfur may inhibit cell growth. The robustness analysis results also showed that the optimized sulfate uptake rate for growth was 0.2 mmol/gDW/h (Additional file 6A). Besides, the experimental results also proved that the addition of Ala, Gly, Asn, Glu, Ser, and Thr (66.7 % of the selected amino acids) could improve DHA production (Fig. 7). Compared to the control group, the addition of Asn led to a 50.6 % increase (9.56 g/L) in DHA production, bringing the DHA content up to 35.0 % of total lipids (Additional file 6B).

Combined with the FBA results, after adding Asn, SR21 could uptake Asn directly from the *in silico* medium, instead of using the pathway to generate Asn starting from Asp. Via adenylosuccinate synthase (EC: 6.3.4.4) and adenylosuccinate lyase (EC: 4.3.2.2), extra Asp was converted into fumarate, enhancing the TCA cycle. As a result, fluxes of two reactions involving acetyl-CoA were also enhanced significantly. For example, the flux of acetyl-CoA supplied by pyruvate dehydrogenase complex (PDC, EC: 1.2.4.1, 2.3.1.12, 1.8.1.4) was increased 28.1 %, and acetyl-CoA flux used for the first step of fatty acid biosynthesis via acetyl-CoA carboxylase (ACC, EC: 6.4.1.2) was also increased 20.9 %. In addition to enhancing acetyl-CoA generation, the addition of Asn also increased NADPH flux for producing DHA. After adding Asn, two fluxes in the pentose phosphate pathway catalyzed by G6PD and PGD both increased 122.6 %. This means adding these amino acids could improve DHA production through enhancing the supply of both acetyl-CoA and NADPH.

### Genetic engineering strategies for improving DHA production by *in silico* overexpression

**Fig. 8:**
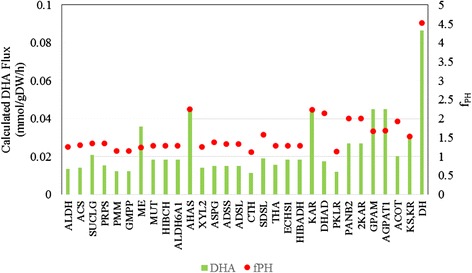
The effect of single gene overexpression on the specific DHA production and f_PH_

MOMA was used to re-calculate the fluxes for the overexpression algorithm. This simulation was carried out based on YE medium. The lower bound of the DHA exchange reaction was set as 0.01 mmol/gDW/h. Then each reaction that had non-zero flux value in the FBA simulation was overexpressed computationally. After finishing the cycle for over-expression, according to equation 1, 32 reactions catalysed by 30 genes were identified as potential targets (Fig. 8, Additional file 7).

These potential targets could be classified into two groups, one of which is directly involved in DHA synthesis while the other is involved in cell growth (Additional file 7). During the biosynthesis of DHA, acetyl-CoA synthetase (ACS, EC: 6.2.1.1) could promote acetyl-CoA generation [34]. Overexpressing ACS led to an increase in DHA production from 0.01 mmol/gDW/h to 0.0143 mmol/gDW/h (an increase of 43.0 %). ME was assumed to be the major supplier of NADPH for fatty acid biosynthesis [35, 36]. When ME was overexpressed *in silico*, DHA production rose to 0.0307 mmol/gDW/h. As a dehydrase/isomerase (DH) involved in the last step of DHA synthesis in the PKS system, 0.0863 mmol/gDW/h DHA was accumulated by overexpressing this gene. Genes involved in cell growth were distributed across many metabolic subsystems, such as carbohydrate metabolism, amino acid metabolism and nucleotide metabolism. For example, phosphomannomutase (PMM, EC: 5.4.2.8) and guanosine diphosphomannose phosphorylase (GMPP, EC: 2.7.7.22) are two genes involved in the synthesis of L-galactose, which is a precursor of the biomass function. The growth rate decreased by 5.7 %, and DHA production increased 22.0 % by overexpressing these two genes. After overexpressing PRPP synthetase (PRPS, EC: 2.7.6.1), fluxes used for the synthesis of AMP increased from 0 to 0.0264 mmol/gDW/h, which led to a 52.0 % improvement in DHA production and an 11.5 % decrease in growth rate.

## Conclusions

A GSMM of *S. limacinum* SR21 for DHA production, named *i*CY1170_DHA, which contained 1769 reactions, 1659 metabolites, and 1170 genes, was successfully reconstructed. Based on glucose and glycerol constraint conditions, the simulated results for growth rate were only 8.5 % and 1.1 % lower than experimental results, respectively. Use of 15 carbon sources and 3 nitrogen sources by SR21 also agreed well with literature reports. Moreover, after the addition of malate and citrate, DHA production increased 24.5 % and 37.1 %, respectively. Furthermore, 9 of 20 amino acids (Ala, Gly, Asn, Asp, Cys, Glu, Gln, Ser, and Thr) were predicted to increase DHA production. According to experimental results, of these 9 amino acids, 6 have been proved to improve DHA production. The addition of Asn could lead a 50.55 % increase in DHA production. Based on MOMA, 30 overexpressed genes, such as those encoding acetyl-CoA synthetase and malic enzyme, were identified as having a positive effect on DHA production.

## Methods

### Reconstruction of the genome-scale metabolic model

The metabolic model of *S. limacinum* was initially reconstructed based on genome annotation information and the metabolic pathway database. First, the genome sequence of *S. limacinum* SR21, which contains 181 scaffolds, was downloaded from the JGI database (http://genome.jgi-psf.org/Aurli1/Aurli1.home.html). Three existing models for *Mortierella alpina* ATCC 32,222 [37], cyanobacterium *Cyanothece* sp. ATCC 51,142 [38], and *Arabidopsis thaliana* [39] were used as reference models, based on protein homology (identity ≥ 40 %, e-value ≤ 1E-30) [40]. KEGG Ontology (KO) and Gene Ontology (GO) identifiers were used to additionally infer reactions that could not be found in the reference strains. To refine the draft model, CELLO [41] and WoLFPSORT [42] were used to determine subcellular compartmentalization. The MetaCyc [43] and BioPath [44] databases were used to judge reaction direction and reversibility. Transport information was obtained by cross-referencing BLATSp searches and the Transporter Classification Database (TCDB) [45].

### Biomass composition

Knowledge of the cellular biomass composition is an important prerequisite for the *in silico* flux analysis, especially during the exponential growth phase, where the primary cellular objective is to maximize growth. The cellular composition of *S. limacinum* SR21 consists of lipids, proteins, carbohydrates, ash, and nucleic acids [46]. The lipid composition was obtained from previous publications on *S. limacinum* [47]. Amino acid and metal ion compositions were determined according to Pyle’s report [46]. The overall DNA and RNA compositions were assumed to be the same as cyanobacterium *Cyanothece* sp. ATCC 51,142 [38] since no data were available on *S. limacinum*. The individual weights of nucleotides in the DNA and RNA were calculated based on the genome sequence of *S. limacinum*, in which the G + C content accounts for 45.17 %. The cell wall composition of *S. limacinum* was assumed to be the same as *S. aggregatum* [48]. Detailed information about biomass composition can found in Additional file 8.

### Constraints-based flux analysis

In order to perform *in silico* simulations, and to predict the metabolic characteristics of *S. limacinum*, constraints-based flux analysis, including flux balance analysis (FBA) [49], was carried out under the assumption of a pseudo-steady state. For growth simulation, the biomass equation was set as the objective function. A complex medium, named yeast extract (YE) medium, which contains the basic elements and 20 amino acids, was used for simulation. The glucose uptake rate was 1.4 mmol/gDW/h according to experimental results [24], and all amino acid maximum uptake rates were set as 0.1 mmol/gDW/h [23]. When glycerol was used as the carbon source, its uptake rate was set as 1.6 mmol/gDW/h [25]. In addition to the YE medium, a minimal glucose (MG) medium that did not contain any amino acids was used for different carbon or nitrogen simulations.

The overexpression algorithm involves five steps [23]. (1) We imposed a DHA production flux of 0.01 mmol/gDW/h, which was higher than the wild-type model production (0.00021 mmol/gDW/h). (2) Flux for each reaction was calculated based on the YE medium. (3) A two-fold flux amplification was imposed for biochemical reactions with non-zero flux (to simulate the effect of gene overexpression). (4) Minimization of metabolic adjustment (MOMA) [50] was used to solve the overexpression problem. (5) An overexpressed target that delivers higher DHA production (over 0.01 mmol/gDW/h) and an f_PH_ value > 1 were identified (Eq. 1), where f_PH_ is the product of the specific biomass overexpression rate and the specific DHA overexpression rate. Steps 3 and 4 were iterated for every reaction within the model.

1 $$ {\mathrm{f}}_{\mathrm{PH}}=\left({\mathrm{f}}_{\mathrm{biomass}}\right)\left({\mathrm{f}}_{\mathrm{DHA}}\right)=\left(\frac{{{\mathrm{V}}_{\mathrm{biomass}}}_{,\;\mathrm{over}\operatorname{expression}}}{{{\mathrm{V}}_{\mathrm{biomass}}}_{,\mathrm{wide}}}\right)\;\left(\frac{{\mathrm{V}}_{\mathrm{DHA}}{{}_{,}}_{\mathrm{over} \exp \mathrm{ression}}}{{{\mathrm{V}}_{\mathrm{DHA},}}_{\mathrm{wide}}}\right) $$

In this study, implementation of constraint-based analysis was performed using Cobra Toolbox 2.05 [51] with MATLAB 2012b and Gurobi 5.6.0 optimizer [52].

### Strain, medium, and culture conditions

*Schizochytrium limacinum* SR21 was gifted by Prof Xiaobin Yu. The fermentation medium contained 120 g/L glucose, 10 g/L peptone, 5 g/L yeast extract, and 20 g/L crystal sea salt [32]. Cells were grown in 500-mL Erlenmeyer flasks each containing 50 mL of medium and incubated at 25 °C in an orbital shaker set at 200 rpm.

### Cell density, dry cell weight, and fatty acid analysis

Cell density was calculated from the optical density measured at 660 nm. DCW was determined by transferring a 5-mL cell suspension to a pre-weighed centrifuge tube and then centrifuging at 5000 r/min for 5 min. The cell pellet was then washed twice with distilled water, and dried at 70 °C to constant weight. Fatty acid analysis was accomplished by first harvesting freshly produced cells and freeze drying overnight. The subsequent methods for fatty acid methyl ester (FAME) preparation and gas chromatographic (GC) analysis are the same as reported by Wang [32].

### Ethics

This manuscript does not involve Ethics.

## Additional files

Additional file 1:
**Detailed information about model** ***i*** **CY1170_DHA.** (XML 3178 kb) Additional file 2:
**Essential genes identified by different** ***in silico*** **media.** (XLSX 38 kb) Additional file 3:
**Comparison among three existing models of** ***S. limacinum***, ***M. alpina***, and *Y. lipolytica*.(XLSX 89 kb) Additional file 4:
**The annotation results about PKS proteins.** (XLSX 9 kb) Additional file 5:
**Reactions involving NADPH which have fluxes in YE medium.** (XLSX 12 kb) Additional file 6:
**A: RobustnessAnalysis result of the relationship between sulfate uptake rate and growth rate.** **B:** The gas chromatography analysis result of adding Asn to the medium. (DOCX 25 kb) Additional file 7:
**Potential targets identified by MOMA which could enhance DHA production.** (XLSX 10 kb) Additional file 8:
**Detailed information about the biomass composition of** ***S. limacinum.*** (XLSX 173 kb)

## Abbreviation

DHA: Docosahexaenoic acid
GSMM: Genome-scale metabolic model, PKS, polyketide synthase
PUFA: Polyunsaturated fatty acid
DCW: Dry cell weight
ARA: Arachidonic acid
EPA: Eicosapentaenoic acid
ORFs: Open reading frames
MG: Minimal glucose
YE: Yeast extract
FAS: Fatty acid synthesis
ACP: Acyl carrier protein
KS: 3-ketoacyl synthase
KR: 3-ketoacyl-ACP reductase
ER: Enoyl reducatase
DH: Dehydrase/isomerase
ME: Malic enzyme
G6PD: Glucose-6-phosphate dehydrogenase
PGD: Phosphogluconate dehydrogenase
ACL: Citrate lyase
ACC: Acetyl-CoA carboxylase
MOMA: Minimization of Metabolic Adjustment algorithm
ACS: Acetyl-CoA synthetase
PMM: Phosphomannomutase
GMPP: Guanosine diphosphomannose phosphorylase
PRPS: PRPP synthetase
KO: KEGG Ontology
GO: Gene Ontology
TCDB: Transporter Classification Database
FAME: Fatty acid methyl ester
GC: Gas chromatographic
FBA: Flux balance analysis.
