# Supplementary material for: Reconstruction and analysis of the genome-scale metabolic model of schizochytrium limacinum SR21 for docosahexaenoic acid production
Source: BMC Genomics. 2015 Oct 16;16:799. doi: 10.1186/s12864-015-2042-y (PMC4609125; doi:10.1186/s12864-015-2042-y)
Supplement: Additional file 6: — A: RobustnessAnalysis result of the relationship between sulfate uptake rate and growth rate. B: The gas chromatography analysis result of adding Asn to the medium. (DOCX 25 kb) [file 12864_2015_2042_MOESM6_ESM.docx]

Additional file 6A: Robustness Analysis result of the relationship between sulfate uptake rate and growth rate.


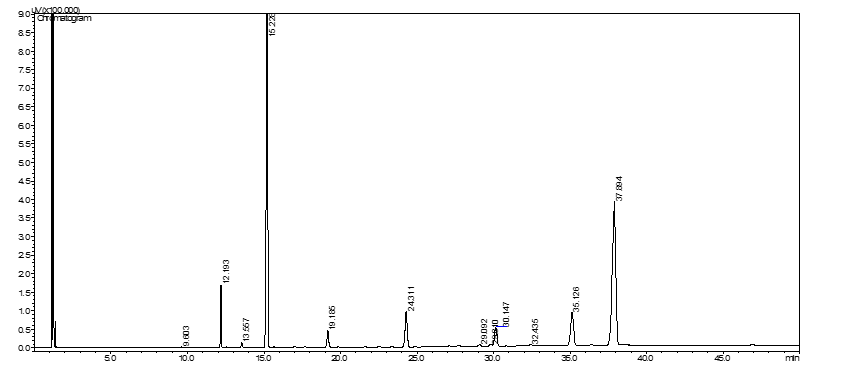


Additional file 6B: The Gas chromatography analysis result of adding Asn in medium.

| Peak# | Ret.Time | Area | Height | Area% | Fatty acid |
| --- | --- | --- | --- | --- | --- |
| 1 | 12.202 | 423374.8 | 115682.0 | 3.1736 | C12：0 |
| 2 | 13.571 | 67577.3 | 14159.3 | 0.5066 | C14：0 |
| 3 | 15.235 | 6828057.7 | 1191441.4 | 51.1833 | C15：0 |
| 4 | 17.048 | 26843.4 | 4138.3 | 0.2012 | C16：0 |
| 5 | 19.205 | 168127.0 | 22036.0 | 1.2603 | C17：0 |
| 6 | 24.297 | 25573.3 | 2672.6 | 0.1917 | C18：0 |
| 7 | 27.741 | 31287.7 | 2688.4 | 0.2345 | C20：0 |
| 8 | 29.115 | 50422.3 | 4891.5 | 0.3780 | C20：5 (EPA) |
| 9 | 29.830 | 25002.3 | 2606.3 | 0.1874 | C22：0 |
| 10 | 32.449 | 45816.5 | 3996.8 | 0.3434 | Unknow |
| 11 | 35.142 | 978973.5 | 70766.9 | 7.3384 | Unknow |
| 12 | 37.889 | 4669336.8 | 281901.5 | 35.0015 | C22：6 (DHA) |
